# Development and validation of pharmaceutical care barriers scale in Chinese hospitals: a cross-sectional survey

**DOI:** 10.3389/fphar.2023.1194901

**Published:** 2023-07-13

**Authors:** Liangjiang Chen, Nan Yang, Yuankai Huang, Xiaoyu Xi

**Affiliations:** ^1^ Research Center of National Drug Policy and Ecosystem, School of International Pharmaceutical Business, China Pharmaceutical University, Nanjing, China; ^2^ West China School of Pharmacy, Sichuan University, Chengdu, China

**Keywords:** barrier, Chinese hospital, clinical pharmacist, pharmaceutical care, scale development

## Abstract

**Introduction:** Chinese hospitals still face various barriers to implementing pharmaceutical care. The quantitative instrument for measuring these barriers in China is scarce. This study aims to develop and validate a scale for measuring barriers to providing pharmaceutical care in Chinese hospitals from the perspective of clinical pharmacists.

**Methods:** The scale was developed based on existing literature and qualitative interviews with 20 experts. The scale was included in a small-range pilot survey and then administered to a validation survey in 31 provinces in China. Exploratory factor analysis was used to identify the structure of the scale. Confirmatory factor analysis was applied to verify the structure of the scale and to validate the scale’s convergent and discriminative validity. Known-group validity was also examined. Cronbach’s alpha examined the internal consistency reliability of the scale.

**Results:** 292 scales were completed and returned for a response rate of 85.6% in the pilot study. Exploratory factor analysis of the scale suggested a five-factor solution (Cognition and attitude, Knowledge and skills, Objective conditions, External cooperation, and Support from managers) accounting for 66.03% of the total variance. 443 scales were sent out in the validation study, with a response rate of 81.0%. Confirmatory factor analysis demonstrated a good fit of the structural model for pharmaceutical care barriers. It showed the scale’s good convergent and discriminative validity (The average variance extracted >0.5 and composite reliability >0.7). The scale could also identify the differences in total score among the clinical pharmacists from different hospital grades (*p* < 0.05). Cronbach’s alpha is between 0.658 and 0.896, indicating good internal consistency.

**Conclusion:** From the perspective of clinical pharmacists, this study has developed a scale to assess obstacles to pharmaceutical care. The scale comprehensively encompasses barriers to clinical pharmacists’ cognitive and ability-related aspects, hindrances encountered in collaborating with other health professionals and patients, and barriers to the working environment. The reliability and validity have been established through verification.

## 1 Introduction

Since Charles Hepler and Linda Strand systemically introduced the concept of pharmaceutical care in the 1990s (Hepler and Strand, 1990), pharmacists have been gradually integrated into the clinical staff as the professionals of pharmacotherapy, by which the role of clinical pharmacist appeared, becoming an indispensable part of medical teams. Up to today, pharmaceutical care is globally performed, benefiting the patients, the physicians, and the pharmacists simultaneously (da Costa et al., 2019).

Similar to other health services, pharmaceutical care is provided depending on mutual collaboration, adequate supply, and efficient utilization of health resources, and thus, it could be hindered by various kinds of barriers (pharmaceutical care barriers) (Onozato et al., 2020). Clinical pharmacists are recognized as the main providers of pharmaceutical care, making their perception of pharmaceutical care barriers both direct and prominent (Zheng et al., 2021). For policymakers and hospital administrators, obtaining feedback from clinical pharmacists provides valuable insights into the pharmaceutical care deficiencies present within hospitals, which is significant for their decision-making on the improvement and development of the pharmaceutical care system, and its proper functioning.

Currently, studies on the pharmaceutical care barriers of most countries focused on community pharmacies, such as those of Europe (Van Mil et al., 2001), Iran (Mehralian et al., 2014), Australia (Culverhouse and Wohlmuth, 2012), Jordan (Elayeh et al., 2017) and New Zealand (Scahill et al., 2009). However, in China, pharmaceutical care in community pharmacies differs from that in hospitals regarding the receiver, the contents, and the standards (Yao et al., 2017), so the conclusions reached by most current studies may not apply to hospitals in China.

Pharmaceutical care barriers in hospitals were only discussed in a few studies (Ngorsuraches and Li, 2006; Lounsbery et al., 2009; Okonta et al., 2012; Katoue et al., 2014; El Hajj et al., 2016; Elsadig et al., 2017; Omar et al., 2020). Due to the diversity of pharmaceutical care barriers around the globe, the appropriate instrument may tremendously affect the robustness and feasibility of the results and conclusions. Detailed information on the instruments used to measure pharmaceutical care barriers, as proposed in these studies, is presented in Table 1. The studies above provided valuable evidence, but given the significant differences between the health system of China and the countries above (Li and Li, 2018), the applicability of those instruments are doubtable. As for the studies on pharmaceutical care barriers in hospitals in China, three studies discussed the factors that may facilitate or obstacle the development of the pharmaceutical care system based on literature and their practical experiences (Wen et al., 2006; Park, 2007; Dong and Wang, 2013). Though these studies generally found that the imperfect laws and regulations, the conventional “drug-orientation” concept of hospital pharmacists, and the clinical incompetence of pharmacists were the major barriers in China, their conclusions were significantly distinct from and incomparable to each other’s and the primary cause of it might be the absence of a valid and applicable instrument for the evaluation of the pharmaceutical care barriers in China.

**TABLE 1:** The basic information of the literature encompassed the instrument for evaluating the pharmaceutical care barriers in hospitals.

Author	Location	Study objective	Basic information of instrument			
			Number of dimensions	Number of items	Dimension classification	Response levels
Ngorsuraches and Li (2006)	Thailand	To examine the comprehension, attitudes, and challenges encountered by pharmacists in Thailand regarding PC.	4	15	Lack of external cooperation, Lack of knowledge and skills, Lack of initiatives, Lack of resources	5-point Likert scale
Lounsbery et al. (2009)	United States	To assess pharmacists’ actual and perceived barriers to implementing MTM services in the outpatient setting and to assess demographic and other factors associated with identified barriers	7	30	Components of MTM, Pharmacist concerns, Interprofessional relationships, Patient care, Management, Compensation	5-point Likert scale
Okonta et al. (2012)	Nigeria	To identify the possible barriers to the implementation of PC among community and hospital pharmacists in Nigeria	4	16	Patient factors, Pharmacist factors, Environmental factors, Monetary factors	5-point Likert scale
Katoue et al. (2014)	Kuwait	To investigate hospital pharmacists’ attitudes towards pharmaceutical care, perceptions of their preparedness to provide pharmaceutical care, and the barriers to its implementation in Kuwait	0	17	No categorization	5-point Likert scale
El Hajj et al. (2016)	Qatar	To examine the extent of PC practice and the barriers to PC provision as perceived by Qatar pharmacists and to assess their level of understanding of PC and their attitudes about PC provision	8	28	Lack of access to patient data, Lack of interaction with patients and healthcare providers, Lack of support from external partners, Societal barriers, Lack of knowledge and skills, Lack of initiatives, Lack of space and time, Lack of resources	5-point Likert scale
Elsadig et al. (2017)	Sudan	To explore the role and challenges facing the clinical pharmacists of Sudan	0	15	No categorization	5-point Likert scale
Omar et al. (2020)	Qatar	To determine perceptions and expectations of healthcare providers toward the clinical pharmacy services at the National Center for Cancer Care and Research	0	8	No categorization	5-point Likert scale
Kopciuch et al. (2021)	Poland	To evaluate the pharmacists’ attitudes towards practice in, and knowledge of PC in Poland and to identify the barriers in PC provision	0	5	No categorization	Yes/No

^a^ PC, pharmaceutical care; MTM, medication therapy management.

^b^ “No categorization” means that the author did not organize the instrument into specific dimensions but instead presented the individual items.

^c^ “5-point Likert scale” indicates that the author used a rating system with five levels to measure the importance of each item. The scale ranges from 1 to 5, representing responses from “strongly disagree” to “strongly agree.”

Compared with the health care system in developed countries, those in developing countries such as China are still in an early stage of development. A considerable amount of health resources was grossly underused due to frequent occurrence of irrational drug use (Mao et al., 2015), such as arbitrary changes in drug dosage, abuse of antibiotics and injections (Wang et al., 2014), pointless combined use of drugs (Guan et al., 2019), etc. In China, healthcare institutions, especially hospitals, are the principal setting of drug use or drug information obtainment, making it essential to improve the pharmaceutical care system, aiming at optimal drug use. In the past 2 decades, the Chinese government has issued several policies concerning the construction of the pharmaceutical care system in Chinese public-based hospitals (Guo et al., 2020). Though China has made remarkable achievements, a noticeable gap in the pharmaceutical care quality remains between China and other developed countries, which could be minimized by identifying and eliminating the pharmaceutical care barriers.

**FIGURE 1:**
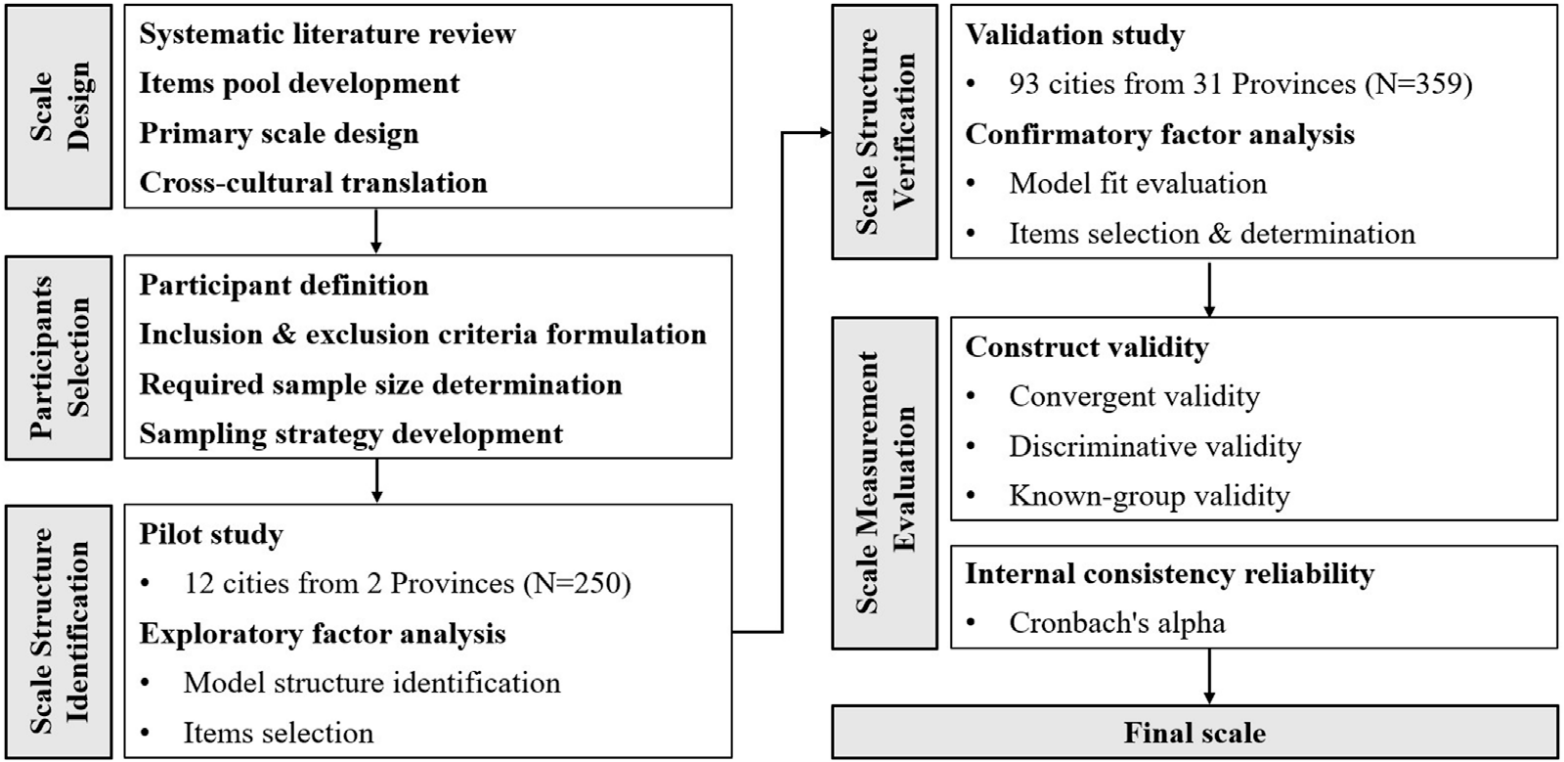
Phases in the development of the PCBS-CH.

This study aims to develop and validate the Pharmaceutical Care Barriers Scale in Chinese Hospitals (PCBS-CH), an instrument to measure barriers to providing pharmaceutical care in Chinese hospitals from the perspective of clinical pharmacists. The flow of this study is shown in Figure 1.

## 2 Methods

### 2.1 Development of the instrument

**FIGURE 2:**
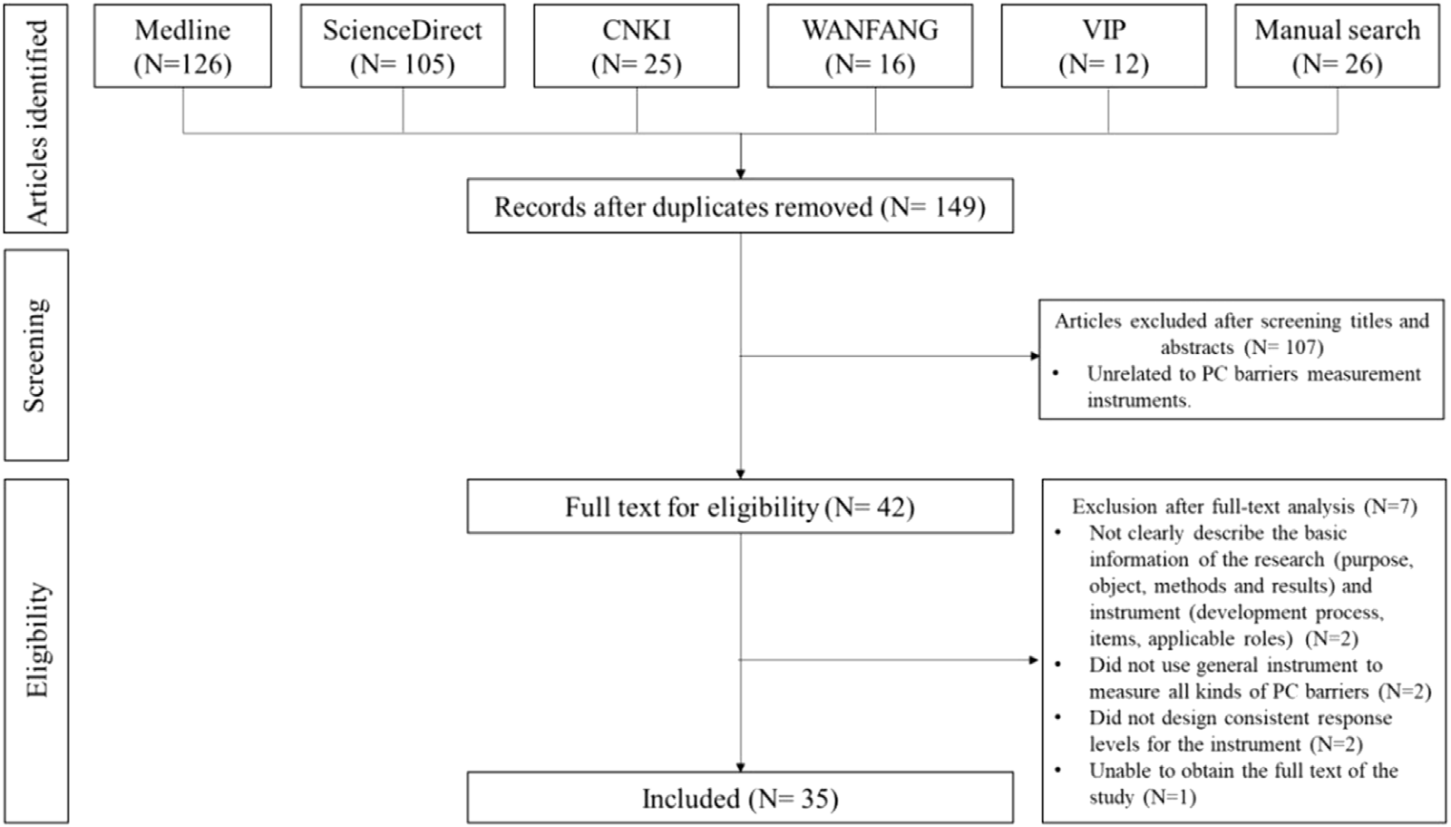
Flow diagram of systematic review.

This study first summarized pharmaceutical care barriers based on a systematic literature review, and an initial pool consisting of the pharmaceutical care barriers was then developed. Related literature was retrieved from Medline, ScienceDirect, China National Knowledge Infrastructure (CNKI), Chinese Scientific Journal Database (VIP) and Wanfang database (WANFANG) during database establishment to Jan. 2019, using “Hospital”, “Medical Institution”, “community pharmacy”, “Pharmaceutical Care”, “Pharmacy Service”, “Pharmaceutical Service”, “Barrier”, “Restriction” and “Obstacle” as keywords. Thirty-five pieces of literature were included after a two-step screening process performed by two researchers. The complete search process is available in Figure 2. All the items were extracted by the researchers and included in the initial pool.

According to the guideline for questionnaire design and scale development (Watson et al., 1988), the primary English scale (Version 1), including 37 items, was developed based on the initial pool. The items comprehensively encompass barriers related to the clinical pharmacists’ cognitive and ability-related aspects, impediments encountered in collaborating with other health professionals and patients, and barriers linked to the working environment. Version 1 was then translated and revised by two translation groups. To ensure the semantical consistency of the original and the translated items (Beaton et al., 2002), a forward translation group was formed by two clinical pharmacists who were fluent in English to translate the Version 1 independently and developed the first Chinese version scale (C-Version 1) while two clinical pharmacists blind to the scales developed the back-translation group to translate C-Version 1 into English (E-C-Version 1). The authors of the primary English scale (Version 1) reviewed and compared E-C-Version 1 and Version 1. To ensure the semantic equivalency, the items of E-C-Version 1 whose concordance rate was less than 70% should be repeatedly translated and back-translated to form the second Chinese version scale (Version 2). Then, experts from a cross-cultural perspective should examine version 2, and the third Chinese version scale was developed (Version 3). Version 3 was sent to an expert panel, including 5 heads of hospital pharmacy departments, 5 front-line clinical pharmacists, 5 staff of pharmaceutical care-related government departments, and 5 university professors engaged in pharmaceutical care-related research from the east, central, and west of China. Experts were required to screen out items confirming China’s national conditions, merge similar items, exclude minor items, and add missing items to synthesize the fourth Chinese version scale (Version 4). Finally, the research team conducted a three-round focus group discussion in February 2019. A total of 10 clinical pharmacists who had participated in the practice of pharmaceutical care were included randomly in each round to revising the difficult-to-understand and ambiguous items of Version 4, and the primary scale for the pilot survey was developed (Version 5). The translation processes can be found in Table 2.

**TABLE 2:** The translation processes.

Process	Decision	Result
Forward translation	Two independent translators (CP)	C-Version 1
	Translate Version 1 into two Chinese version	
	Reconciliation	
Backward translation	Another two independent translators (CP)	E-C-Version 1
	Translate C-Version 1 into 2 English version	
	Reconciliation	
Concordance rate checking	Authors of Version 1	Version 2
	Compare E-C-Version 1 with Version 1	
	Translate and back-translate repeatedly	
Expert verification	Twenty experts	Version 4
	Test cross-cultural adaptation to form Version 3	
	Discussion and amendment	
Focus group discussion	Ten CPs	Version 5
	Fill the scale (Version 4)	
	Discuss their understanding of each item	
	Amendment	

^a^ CP, clinical pharmacist.

^b^ The Version 1 including 37 items.

^c^ The Version 5 included 27 items.

The primary version scale (Version 5) included 27 items, which basically covered all barriers to performing pharmaceutical care in Chinese hospitals. A 5-point Likert scale, where 1 = strongly disagree and 5 = strongly agree, was used to elicit responses to 27 possible barriers to providing pharmaceutical care. The scale is available online as Supplementary Table S1. The sum of each item calculated the total score. Meanwhile, respondents need to fill out a basic information questionnaire to collect data on sex, age, education, and years of practicing as a clinical pharmacist.

### 2.2 Participants

First-line clinical pharmacists from all parts of China were recruited in this survey. The inclusion criteria for the sample were: 1) Be a full-time clinical pharmacist working in the sampled hospital. 2) Have been working for more than 1 year. 3) Be willing to sign the informed consent. The exclusion criteria were: 1) Clinical pharmacist on vacation or training. 2) Clinical pharmacist who had not provided pharmaceutical care.

Based on the principle that the sample size should be 5 to 10 times of the number of items (27 items) to ensure the results of Exploratory Factor Analysis (EFA) are more precise estimates of factor loadings and are also more stable (Bentler et al., 1987; Gorsuch, 2013), assuming the missing rate of the scale response (20%), a minimum sample size of 169 was estimated to be sufficient for the pilot study, while a sample size of 338 would be even more desirable.

A multi-stage sampling strategy was adopted to ensure sample representativeness of the validation study: 1) The sample covered all 31 provincial administrative regions in mainland China. 2) Cities in each provincial administrative region were divided into three groups according to their 2018 *per capita* gross domestic product, which is associated with the city’s medical resources possession, such as the quality of healthcare techniques, thereby generating 93 groups. 1 city or district was selected using the random number method in each group, thereby 93 cities or districts were selected. 3) In each selected city or district, 1 secondary hospital and 1 tertiary hospital were surveyed by convenience based on the hospital administrators’ permission to conduct the survey, with the hospital level verified by consulting the hospital information tool by the National Health Commission of China. This ensured that 186 hospitals would be selected. 4) In each surveyed hospital, 1 to 2 clinical pharmacists in secondary hospitals and 2 to 4 clinical pharmacists in tertiary hospitals were selected by convenience, recommended by the hospital administrator(s), or another participant who completed the survey. Overall, at least 279 scales should be distributed during the validation study, and the number of total scales should not exceed 558.

### 2.3 Pilot study

A three-round pilot study was conducted in 64 secondary hospitals and 71 tertiary hospitals in 6 cities of Jiangsu Province and 6 cities of Anhui Province, from March to April 2019. The investigator explained the intention to the clinical pharmacists to sign an informed consent, and an appropriate time and place was determined for a face-to-face interview. Researchers collated questions from investigators and respondents in the pilot study, such as respondents’ comments, unanswered items, the time spent answering each item, and the total time required to accomplish the scale. EFA was performed on the pilot study data to obtain a general model of dimensions of pharmaceutical care barriers. Researchers revised the scale again according to the feedback from the pilot study. The eventual scale for the validation study was divided into 5 dimensions, including 21 items.

### 2.4 Validation study

A total of 31 undergraduate students majoring in pharmacy or clinical pharmacy were recruited as data collectors and trained. The validation study was carried out from July to September 2019. After obtaining the hospital administrators’ consent, during the non-working hours of the hospital, the investigators asked the sampled clinical pharmacists for their basic information to determine whether they met the inclusion criteria and then provided the eligible clinical pharmacists with the purposes, the contents and the requirements of our survey and confirmed their willingness to participate in again. Those willing to participate signed the consent form and decided the time and a quiet place for the survey with the investigators. The investigator stated the requirements for each item, the description of the items, and the options of each item and recorded the oral answers of the respondents through an online survey system. After the interview, the data were immediately uploaded to the researcher’s server and converted into a database file that the data analysis software could recognize. A team of 5 postgraduate students was recruited to review the uploaded data and immediately return the documents with errors or damaged data for correction through return visits.

### 2.5 Ethical approval

The ethical approval to conduct the pilot survey and validation study was granted by the Ethics Committee of China Pharmaceutical University (Project Number: CPU2019015). Written consent to participate was obtained from each participant before data collection. No sensitive and personal data were recorded, while confidentiality of data was assured during data analysis and reporting.

### 2.6 Statistical analysis

EFA with principal components analysis and varimax rotation was carried out to obtain a general model of the dimension of the pharmaceutical care barriers and determine the formal scale. Confirmatory factor analysis (CFA) was applied to verify the structural validity of the scale and goodness of fit for the model. One-way ANOVA was used to determine the structural validity in a known group. Shapiro-Wilk test and Levene’s test were applied to test the normality and homogeneous variance of the sample before the one-way ANOVA. Internal consistency reliability was examined using Cronbach’s alpha.

For EFA, the KMO value (Kaiser–Meyer–Olkin) is used to determine whether factor analysis is possible. The closer to 1 the KMO is, the more suitable the variable for factor analysis is (Batistelli Vignola and Tucci, 2014). The following criteria are mainly used when selecting the items: 1) the load value of a variable on a factor is greater than 0.5; 2) the cross load value between variables is low; 3) the characteristic value of the variable is greater than 1; 4) items with the same factor have the same meaning (Fabrigar et al., 1999). Questionnaires that are more reliable possess a higher value of Cronbach’s alpha. A value of 0.7 is regarded as an acceptable value (Peill et al., 2022). For CFA, the absolute fitting index, relative fitting index, and goodness-of-fit index were used to check the model’s fit (Alamer and Marsh, 2022). The average variance extracted (AVE) > 0.5, Composite Reliability (CR) > 0.7 (Bacon et al., 1995) implies good aggregation validity; The AVE root value of each factor is greater than the maximum value of the correlation coefficient, indicating good discriminative validity (Dash and Paul, 2021). For one-way ANOVA, *p* < 0.05 represents a significant difference (Lix et al., 1996).

The CFA used IBM SPSS AMOS version 24.0 (IBM SPSS Statistics, Armonk, NY). All other statistical analyses were performed using IBM SPSS statistics version 26.0 (SPSS Inc., Chicago, IL).

## 3 Results

### 3.1 Participant characteristics

The pilot study involved the participation of 292 clinical pharmacists, with 250 valid scales recovered, resulting in a recovery rate of 85.6%. Out of the 443 scales distributed in the validation study, we received a response rate of 81.0%, yielding 359 effective scales. Females accounted for more than half of the study population. The respondents’ age, professional title, and education were concentrated in the bracket of 30–39, intermediate titles, and undergraduates. The demographic information is consistent with the previous survey of pharmaceutical care in China (Xi et al., 2017), except for the distribution of academic qualifications, which may be attributed to a convenient sampling method to select hospitals and clinical pharmacists (Table 3).

**TABLE 3:** Sociodemographic characteristics of respondents of the pilot study and validation study.

Item	Pilot study (N = 250)				Validation study (N = 359)			
	Sec-CPs		Ter-CPs		Sec-CPs		Ter-CPs	
	N	%	N	%	N	%	N	%
Gender								
Male	34	29.82	48	35.29	36	31.03	89	36.63
Female	80	70.18	88	64.71	80	68.97	154	63.37
Total	114	100.00	136	100.00	116	100.00	243	100.00
Age (years)								
20–29	28	24.56	24	17.65	24	20.69	43	17.70
30–39	82	71.93	109	80.15	58	50.00	139	57.20
40–49	2	1.75	3	2.21	29	25.00	49	20.16
≥50	2	1.75	0	0.00	5	4.31	12	4.94
Total	114	100.00	136	100.00	116	100.00	243	100.00
Professional Title								
Pharmacist	57	50.00	64	47.06	53	45.69	76	31.28
Pharmacist-in-charge	51	44.74	70	51.47	53	45.69	133	54.73
Associate professor of pharmacy	6	5.26	2	1.47	9	7.76	30	12.35
Professor of pharmacy	0	0.00	0	0.00	1	0.86	4	1.65
Total	114	100.00	136	100.00	116	100.00	243	100.00
Academic degree								
Lower than Bachelor degree	13	11.40	12	8.82	22	18.97	23	9.47
Bachelor degree	102	89.47	115	84.56	101	87.07	206	84.77
Graduate degree	29	25.44	65	47.79	24	20.69	104	42.80
Doctoral degree	0	0.00	6	4.41	0	0.00	6	2.47
Total	114	100.00	136	100.00	116	100.00	243	100.00
Years working in hospital								
1–9	30	26.32	108	79.41	56	48.28	142	58.44
10–19	72	63.16	26	19.12	44	37.93	68	27.98
≥20	12	10.53	2	1.47	16	13.79	33	13.58
Total	114	100.00	136	100.00	116	100.00	243	100.00

^a^ Sec-CPs, clinical pharmacists working in secondary hospital; Ter-CPs, clinical pharmacists working in tertiary hospital

^b^ In China, since 2011, clinical pharmacists have been mandated to possess a bachelor’s degree or higher. Nevertheless, some in-service clinical pharmacists with limited education were allowed to continue their practice after receiving standardized training.

### 3.2 Exploratory factor analysis

EFA was adopted to identify the structure of the PCBS-CH. For EFA, The KMO value was 0.89, and Bartlett’s test of sphericity reached statistical significance (chi-square = 2,615.41, *p* < 0.05), supporting the factorability of the correlation matrix. According to the above criteria (see statistical analysis), 6 items were eliminated after factor extraction, and the factor loading of each item reached the standard. Table 4 presents the items with their loadings in each factor. The eigenvalue of each factor was greater than 1, and the cumulative interpretation rate was 66.026%. The factors were labeled after closely examining the content of the items constituting each factor. Factor 1, termed “cognition and attitude,” comprised 3 items and explained 5.091% of the variance. Factor 2, labeled “knowledge and skills,” consisted of 4 items, accounting for 7.978%. Factor 3, denoted as “objective conditions,” encompassed 7 items and explained a substantial 37.249%. Factor 4, designated as “external cooperation,’” comprised 3 items, accounting for 7.093%. Lastly, Factor 5, labeled “support from managers,” comprised 4 items and explained 8.615% of the variance.

**TABLE 4:** Rotated Factor Loadings for the scale.

Factor	Item	Factor loading
Factor 1	1. Lack of understanding of the components of pharmaceutical care	0.678
	2. Inappropriate attitude of pharmacists toward pharmaceutical care	0.867
	3. Lack of confidence for pharmaceutical care development	0.586
Factor 2	5. Lack of communication skills	0.652
	6. Lack of knowledge in clinical pharmacy	0.820
	7. Lack of knowledge in clinical medicine	0.770
	8. Lack of electronic information technology and document retrieval skills	0.705
Factor 3	10. Lack of an electronic management system of pharmaceutical care	0.653
	11. Lack of additional staffing (pharmacist, technician, or support staff)	0.666
	12. Lack of rules and regulations of pharmaceutical care practice	0.537
	13. Lack of physical space for pharmaceutical care provision	0.720
	14. Lack of time for pharmaceutical care provision	0.580
	15. Lack of an electronic information system and prescription evaluation system	0.758
	16. Lack of an efficient and standardized documentation system	0.723
Factor 4	18. Lack of communication with doctors and their support	0.856
	19. Lack of communication with other medical service staff and their support	0.874
	20. Lack of communication with patients and their support	0.792
Factor 5	23. Lack of opportunities for continuing education	0.808
	24. Lack of time for continuing education	0.697
	25. Lack of support from hospital leaders	0.567
	26. Lack of support from upper management	0.688

^a^ Factor1 = Cognition and attitude, Factor2 = Knowledge and skills, Factor3 = Objective conditions, Factor4 = External cooperation, Factor5 = Support from managers.

^b^ Principal components analysis with varimax rotation was employed to conduct exploratory factor analysis.

^c^ After factor extraction, item 4, 9, 17, 21, 22, and 27 were removed from the scale.

### 3.3 Confirmatory factor analysis

**FIGURE 3:**
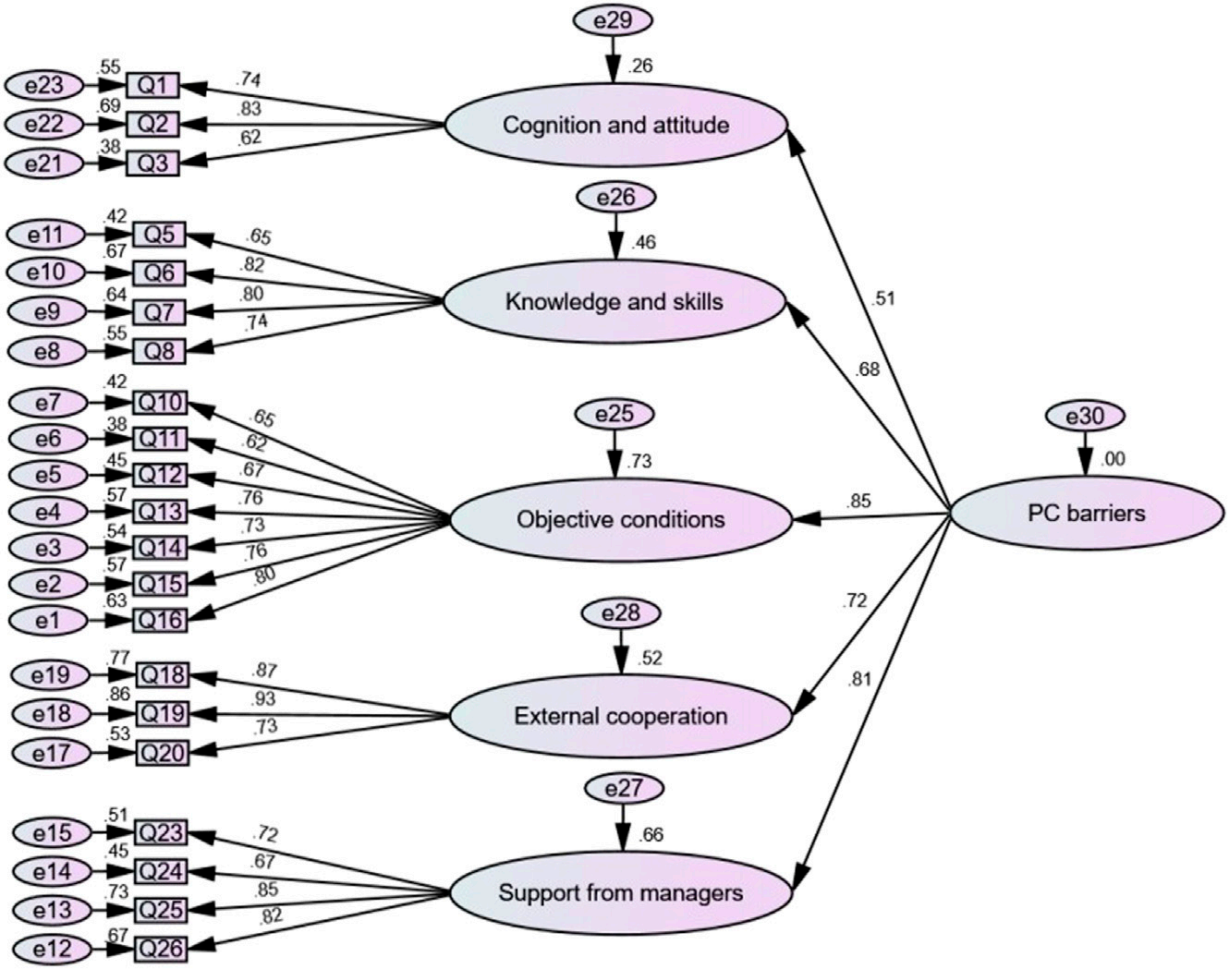
Structural model of pharmaceutical care barries

CFA with the method of maximum likelihood was applied to the sample of the validation study (n = 359) and resulted in the same five-factor structure as above. Figure 3 shows the structural model with the individual items of the PCBS-CH and standardized coefficients of each path; the highest and the lowest coefficients were related to “Objective conditions” and “Cognition and attitude”. The path diagram provides a clear representation of the factor structure of the theoretical concept, with each variable loading exclusively on one factor. In short, Figure 3 indicates that the structural model, consisting of 21 items and organized into five factors, serves as an appropriate measure of pharmaceutical care barriers in the present sample.

The structural model showed an overall satisfactory fit in the CFA, as indicated by the following values for the goodness of fit indicators: SRMR and RMSEA were 0.068 and 0.072, respectively, below the threshold of 0.08. TLI, CFI, and IFI values were 0.902, 0.915, and 0.915, respectively, approaching the desired level of 0.9. PNFI and PGFI values were 0.766 and 0.698, respectively, exceeding the 0.50 threshold. In addition, the χ^2^/df value was 2.844, indicating a good fit (less than 3). Overall, all indicators met the requirements for the goodness of fit, which implied that the developed five-factor PCBS-CH had enough validity to show a relationship between latent and visible variables amongst the Chinese clinical pharmacist population (Table 5).

**TABLE 5:** Goodness-of fit measures.

	Absolute fit indicators		Incremental fit indicators			Goodness of fit index		
Fitness indicator	SRMR	RMSEA	TLI	CFI	IFI	χ^2^/df	PNFI	PGFI
Validation value	0.068	0.072	0.902	0.915	0.915	2.844	0.766	0.698
Suggested criteria	<0.08	<0.08	>0.9	>0.9	>0.9	<3	>0.5	>0.5

^a^ SRMR: standardized root-mean-square residual, RMSEA: root mean square error of approximation, TLI, tucker lewis index; CFI, comparative fit index; IFI, incremental fit index, χ^2^ = Chi-square, df = Degree of freedom, PNFI, parsimonious normed fit index; PGFI, parsimonious goodness of fit index.

^b^ The model’s fit was assessed using absolute fitting index, relative fitting index, and goodness-of-fit index.

^c^ The validation values should ideally demonstrate close proximity to or surpass the suggested criteria, as per theoretical expectations.

### 3.4 Convergent and discriminative validity

For CFA, AVE >0.5 and CR > 0.7 for all factors indicated good convergent validity. The square root of AVE of each factor was all larger than the absolute value of the correlation coefficient between factors, indicating that the internal correlation was greater than the external correlation, reflecting good discriminative validity (Table 6).

**TABLE 6:** Aggregation and discriminant validity index of pharmaceutical care barriers scale.

	AVE	CR	Factor1	Factor2	Factor3	Factor4	Factor5
Factor1	0.543	0.857	0.737^c^				
Factor2	0.570	0.901	0.346^b^	0.755^c^			
Factor3	0.509	0.878	0.435^b^	0.577^b^	0.714^c^		
Factor4	0.717	0.931	0.367^b^	0.487^b^	0.613^b^	0.847^c^	
Factor5	0.592	0.909	0.414^b^	0.549^b^	0.691^b^	0.583^b^	0.769^c^

^a^ Factor1 = Cognition and attitude, Factor2 = Knowledge and skills, Factor3 = Objective conditions, Factor4 = External cooperation, Factor5 = Support from managers, AVE, average variance extracted; CR, composite reliability.

^b^ Absolute value of the correlation coefficient between factors.

^c^ Square root of AVE.

### 3.5 Known-group validity

Known-group validity was also examined regarding the difference between respondents’ total score from different hospital grades. The total score of each subgroup was expressed in the form of mean ± standard deviation: secondary hospital (55.02 ± 12.43), tertiary hospital (49.98 ± 12.77). The difference between various categories of ‘hospital grade’ was statistically significant (F (1,357) = 12.431, *p* < 0.05).

### 3.6 Internal consistency reliability

Cronbach’s alpha tests yielded favorable results for the PCBS-CH. The overall alpha coefficient was 0.865, while the individual dimensions demonstrated internal consistency reliability with Cronbach’s alpha values of 0.658, 0.830, 0.862, 0.896, and 0.788, respectively. These results indicate acceptable levels of internal consistency reliability for both the instrument and its sub-dimensions.

## 4 Discussion

This study developed an instrument for measuring the pharmaceutical care barriers in hospitals in China. Under this topic, PCBS-CH is the first instrument developed and validated using psychometric methods. The results proved the good reliability and construct validity of the 21-item scale. PCBS-CH is an applicable instrument that allows policymakers and researchers to measure the pharmaceutical care barriers in hospitals in China on a unified scale. This evidence can be helpful for them to fully understand all the types of existing barriers, accurately assess the importance of different barriers, and formulate strategies to eliminate barriers in the pharmaceutical care practice.

A systemic approach identified five dimensions of pharmaceutical care barriers in hospitals in China and then validated them. Among them, the “Cognition and attitude” combined the dimension “Components of MTM’ and “Attitude and vision”, which were mentioned in the instrument from US and Europe (Van Mil et al., 2001; Lounsbery et al., 2009). “Knowledge and skills” combined the dimensions “Lack of knowledge and skills”, “Skills” and ‘Interprofessional relationships” which was mentioned in the instrument from Thailand, Europe and US (Van Mil et al., 2001; Ngorsuraches and Li, 2006; Lounsbery et al., 2009). “Objective conditions” combined the “Lack of resources” and “Resources” dimensions in the Thai and European instrument (Van Mil et al., 2001; Ngorsuraches and Li, 2006). “External cooperation” and “Support from managers” were respectively similar to “Lack of external cooperation” and “management” dimensions referred to in the Thai and American instrument (Ngorsuraches and Li, 2006; Lounsbery et al., 2009). However, the items in dimension “Regulatory and environment” in Iranian scale was excluded into the initial pool (Mehralian et al., 2014), because the Chinese government has gradually attached importance to the development of pharmaceutical care, and there is a good regulatory and legal environment in China (Sun et al., 2008).

EFA indicated that most items were validated to be loaded on the dimension as they were in the primary version scale (Version 5), suggesting that most items were strongly related to the corresponding factor as expected. One exception is the item “Lack of sufficient compensation for pharmaceutical care provision”, of which the factor loading on the factor “objective conditions” was smaller than the acceptable threshold (0.5), indicating that this item had no practical significance, so this item was removed. However, there is a growing call from Chinese clinical pharmacists to introduce ‘pharmaceutical care fees’ to recognize the value of their work (Zhang et al., 2020; Zhang et al., 2021). Although pharmaceutical care does not depend on these fees because there is no specific provision in Chinese law requiring patients to pay for pharmaceutical care, their implementation could increase clinical pharmacists’ motivation to provide higher-quality services. Therefore, in this study, the absence of financial compensation was not considered a barrier but rather an appropriate facilitator and was therefore excluded from the scope of this scale. Furthermore, with the enactment of the Pharmacists Act, the issue of ‘pharmaceutical care fees’ is expected to be adequately addressed.

The item “lack of opportunities for continuing education” and item “lack of time for continuing education” were validated to be loaded on the factor of “Support from managers” rather than “Knowledge and skills” as initially hypothesized. This may be because administrators of hospitals or specific clinical departments in China have great decision-making power on the staffing and in-service education of their subordinates for the convenience and feasibility of personnel management (Penm et al., 2014). Thus, pharmacists tend to believe that the lack of opportunities for continuing education is in the scope of the administrators’ responsibility.

The items 21 and 22 are also noteworthy. Both of them were removed according to EFA because they had no acceptable factor loading on any factor extracted. Besides reflecting the pharmacist’s power to review and modify the prescriptions, these two items seem to represent the degree of the pharmacist’s participation in the clinical decision-making regarding a patient’s medication. Clinical pharmacists in China are required to implement effective interventions in prescriptions audit and drug dispensing according to ‘Management standard of hospital prescription comment’. Therefore, relevant barriers cannot be perceived easily by clinical pharmacists.

Observed differences in total PCBS-CH score between sub-groups of pharacists were consistent with the known differences in the development of pharmaceutical care systems of those sub-groups (Xi et al., 2017), and it validated the construct validity of the PCBS-CH as external evidence. Consistent with the *status quo* of China’s pharmaceutical care system development, clinical pharmacists in tertiary hospitals have significantly lower degrees of perceived barriers than that in secondary hospitals. The average total score was 5 points higher in secondary hospitals, indicating more potential barriers in certain domains. In the clinical application of the scale, PCBS-CH demonstrates enough sensitivity in capturing variations in pharmaceutical care barriers across hospitals, enabling policymakersto identify the source and severity of these barriers accurately. They can then formulate targeted strategies accordingly.

Though the PCBS-CH was designed to measure the pharmaceutical care barriers of hospitals in China, it may have the potential to be applied in community pharmacies or primary healthcare institutions after necessary validations or adaptions. From a global perspective, the development of pharmaceutical care systems in all types of healthcare institutions is universally in the early stage and may be facing common barriers, such as lack of objective conditions and external cooperation.

Several limitations to the study should be noted: firstly, owing to limited resources, this study resorted to a convenient sampling approach for selecting hospitals and clinical pharmacists, potentially impacting the representativeness of the samples. Although the study results suggest relative balance across dimensions, it is worth noting that clinical pharmacists with Doctoral degrees were underrepresented in the sample. Therefore, future research should consider quota sampling based on the demographic characteristics of clinical pharmacists to enhance sample representativeness and ensure a more comprehensive evaluation of the population. Secondly, due to time constraints, it was a cross-sectional survey, so the retest reliability cannot be checked. Thus, further longitudinal studies are warranted to test their measurement properties.

## 5 Conclusion

This study developed and validated an instrument (PCBS-CH) for measuring the pharmaceutical care barriers in hospitals in China. This instrument was shown to have good reliability and construct validity. It provided the policymakers and hospital administrators with an applicable and reliable tool for finding the direction for improving the current pharmaceutical care system. This instrument may be suitable for other settings of pharmaceutical care in China, such as community pharmacies or primary healthcare institutions, for which validations and adaptions are needed.

## Data Availability

The original contributions presented in the study are included in the article/Supplementary Material, further inquiries can be directed to the corresponding author.
