# Supplementary material for: Development and validation of pharmaceutical care barriers scale in Chinese hospitals: a cross-sectional survey
Source: Front Pharmacol. 2023 Jul 13;14:1194901. doi: 10.3389/fphar.2023.1194901 (PMC10373295; doi:10.3389/fphar.2023.1194901)
Supplement: Supplementary file 1 [file Table1.docx]

Supplementary Material

Development and Validation of Pharmaceutical Care Barriers Scale in Chinese Hospitals: a Cross-Sectional Survey

**Liangjiang Chen, Nan Yang, Yuankai Huang, Xiaoyu Xi***

*** Correspondence:** Corresponding Author: [xixy@cpu.edu.cn](mailto:xixy@cpu.edu.cn)

**Supplementary** **Table 1.** **The Pharmaceutical Care Barriers Scale.**

Dear Clinical Pharmacist:

Thank you for participating in this survey!

The purpose of this scale is to measure the barriers you faced in your work at this hospital. The survey results will be kept strictly confidential and used only for academic research. Please read each of the following statements and circle the one answer that come closest to your feelings. Thank you for your support and cooperation.

| Item | Strongly disagree | Disagree | Neither agree or disagree | Agree | Strongly agree |
| --- | --- | --- | --- | --- | --- |
| 1. Lack of understanding of the components of pharmaceutical care | 1 | 2 | 3 | 4 | 5 |
| 2. Inappropriate attitude of pharmacists toward pharmaceutical care | 1 | 2 | 3 | 4 | 5 |
| 3. Lack of confidence for pharmaceutical care development | 1 | 2 | 3 | 4 | 5 |
| 4. Lack of enough compensation for pharmaceutical care provision^a^ | 1 | 2 | 3 | 4 | 5 |
| 5. Lack of communication skills | 1 | 2 | 3 | 4 | 5 |
| 6. Lack of knowledge in clinical pharmacy | 1 | 2 | 3 | 4 | 5 |
| 7. Lack of knowledge in clinical medicine | 1 | 2 | 3 | 4 | 5 |
| 8. Lack of electronic information technology and document retrieval skills | 1 | 2 | 3 | 4 | 5 |
| 9. Lack of ability to market pharmaceutical care to patients^a^ | 1 | 2 | 3 | 4 | 5 |
| 10. Lack of an electronic management system of pharmaceutical care | 1 | 2 | 3 | 4 | 5 |
| 11. Lack of additional staffing (pharmacist, technician, or support staff) | 1 | 2 | 3 | 4 | 5 |
| 12. Lack of rules and regulations of pharmaceutical care practice | 1 | 2 | 3 | 4 | 5 |
| 13. Lack of physical space for pharmaceutical care provision | 1 | 2 | 3 | 4 | 5 |
| 14. Lack of time for pharmaceutical care provision | 1 | 2 | 3 | 4 | 5 |
| 15. Lack of an electronic information system and prescription evaluation system | 1 | 2 | 3 | 4 | 5 |
| 16. Lack of an efficient and standardized documentation system | 1 | 2 | 3 | 4 | 5 |
| 17. Lack of recognition as a medical service provider^a^ | 1 | 2 | 3 | 4 | 5 |
| 18. Lack of communication with doctors and their support | 1 | 2 | 3 | 4 | 5 |
| 19. Lack of communication with other medical service staff and their support | 1 | 2 | 3 | 4 | 5 |
| 20. Lack of communication with patients and their support | 1 | 2 | 3 | 4 | 5 |
| 21. Poor access to medical information, including medical records and prescriptions^a^ | 1 | 2 | 3 | 4 | 5 |
| 22. Lack of ability to modify drug therapy for patients^a^ | 1 | 2 | 3 | 4 | 5 |
| 23. Lack of opportunities for continuing education | 1 | 2 | 3 | 4 | 5 |
| 24. Lack of time for continuing education | 1 | 2 | 3 | 4 | 5 |
| 25. Lack of support from hospital leaders | 1 | 2 | 3 | 4 | 5 |
| 26. Lack of support from upper management | 1 | 2 | 3 | 4 | 5 |
| 27. Lack of support from pharmacists’ law^a^ | 1 | 2 | 3 | 4 | 5 |

^a^Deleted from the scale used for validation study.
